# Analysis of Genome Survey Sequences and SSR Marker Development for Siamese Mud Carp, *Henicorhynchus siamensis*, Using 454 Pyrosequencing

**DOI:** 10.3390/ijms130910807

**Published:** 2012-08-29

**Authors:** Feni Iranawati, Hyungtaek Jung, Vincent Chand, David A. Hurwood, Peter B. Mather

**Affiliations:** Earth, Environmental and Biological Sciences, Science and Engineering Faculty, Queensland University of Technology, Brisbane, Queensland 4000, Australia; E-Mails: v.chand@qut.edu.au (V.C.); d.hurwood@qut.edu.au (D.A.H.); p.mather@qut.edu.au (P.B.M.)

**Keywords:** *Henichorynchus siamensis*, 454 pyrosequencing, SSR marker

## Abstract

Siamese mud carp (*Henichorynchus siamensis*) is a freshwater teleost of high economic importance in the Mekong River Basin. However, genetic data relevant for delineating wild stocks for management purposes currently are limited for this species. Here, we used 454 pyrosequencing to generate a partial genome survey sequence (GSS) dataset to develop simple sequence repeat (SSR) markers from *H. siamensis* genomic DNA. Data generated included a total of 65,954 sequence reads with average length of 264 nucleotides, of which 2.79% contain SSR motifs. Based on GSS-BLASTx results, 10.5% of contigs and 8.1% singletons possessed significant similarity (*E* value < 10^−5^) with the majority matching well to reported fish sequences. KEGG analysis identified several metabolic pathways that provide insights into specific potential roles and functions of sequences involved in molecular processes in *H. siamensis*. Top protein domains detected included reverse transcriptase and the top putative functional transcript identified was an ORF2-encoded protein. One thousand eight hundred and thirty seven sequences containing SSR motifs were identified, of which 422 qualified for primer design and eight polymorphic loci have been tested with average observed and expected heterozygosity estimated at 0.75 and 0.83, respectively. Regardless of their relative levels of polymorphism and heterozygosity, microsatellite loci developed here are suitable for further population genetic studies in *H. siamensis* and may also be applicable to other related taxa.

## 1. Introduction

Siamese mud carp (*Henichorynchus siamensis*) is a phytoplankton feeding, freshwater carp species that occurs naturally in bottom to mid water depths widely across the Mekong River Basin (MRB) and in some other drainages in South East Asia [1]. While categorized as a small cyprinid (up to 20 cm), *H. siamensis* numbers are so abundant that at certain times of the year in the lower MRB they constitute a significant percentage of the total fish catch in the river. This species is morphologically very similar to *H. lobatus* and based on several catch reports, fisherman find them hard to distinguish and so they are classified simply as small cyprinids in catch data, although densities of the two species vary widely in different areas [2]. The two *Henichorynchus* species contributed more than 50% to the total bag-net fish catch in Cambodia inland waters in 1994 [3] and approximately 50% in the Khone Falls region [4]. Although annual fish catch trends in the MRB are likely to increase, compared with catch effort, total production in 2000 was less than half of that obtained in 1994 [5], a result that is a warning about potential threats to Mekong River fisheries and aquatic biodiversity across the region.

A recent analysis of *H. siamensis* wild stock structure in the MRB using mtDNA markers identified 3 discrete stocks [6], while in contrast, for the closely related *H. lobatus*, a single panmictic stock was detected across the basin, with the exception of a sample from the Mun River (a tributary of the Mekong) that was highly divergent and indicated a population that had been evolving independently for a long period of time [7]. These two genetic studies suggest that previous ecological reports [4] of long distance migrations by both species, both up and down stream and in the main Mekong River channel may be incorrect. Different wild population structures in these two species indicate that they may have had different evolutionary histories in the MRB. Discrete populations of mud carp in the MRB that apparently exchange genes at different geographical scales suggest multiple management units.

A major limitation of these studies however, was that they only estimated female gene flow and hence provided only partial resolution of the structure of sampled wild populations [8]. The problem with only assessing female gene flow was highlighted further by the fact that a single individual from the Mekong River clade was sampled in the Mun River, indicating that both genetic groups may co-exist to some extent. Using a molecular marker that is inherited maternally does not provide insight into whether individuals from divergent lineages actually interbreed in the wild. Furthermore, because mtDNA is a single genetic locus, it may have insufficient power to fully resolve fine scale population structure where it exists [9]. A multi-locus nuclear marker (e.g., SSR) approach in parallel with mtDNA analysis can address this problem and provide a comprehensive assessment of the population structure present [9,10]. Effective management of these important fisheries resources will require a detailed understanding of the geographical scale at which discrete wild stocks are present to guarantee sustainable harvesting into the future.

Microsatellites or SSRs representing tandem repeated short DNA sequences (1–8 bases) are scattered widely and randomly across prokaryotic and eukaryote genomes, can be scored easily [11] and conform to the laws of Mendelian inheritance [12]. As they are nuclear DNA markers, each diploid individual will carry two copies and they are inherited in a co-dominant fashion, so they are useful for assessing population differentiation in gene flow studies due to their high allelic variation. In general, SSR loci typically encode large allelic variation (often >10 alleles) per locus and have potential for cross amplification in closely related taxa [13–15]. Fast evolution rate in SSRs is an advantage for detecting the effects of recent demographic events [16] so SSR markers have become the preferred tool for application in fisheries stock management, population analyses, and biodiversity preservation programs due to their ability to detect differences between closely related populations and their efficiency for revealing extensive allelic variation [17,18].

SSR marker development can be problematic however, in many non-model species because of the high cost and time required for library construction using the traditional approach [14,15]. This can now be overcome using next generation sequencing techniques (NGST), for example 454 pyrosequencing. Significant random genomic DNA fragment (genome survey sequence or GSS) and large expressed sequence tag (EST) data sets can be generated from Roche 454 pyrosequencing and can be applied to identify new genes and data resources including for SSR and single nucleotide polymorphism (SNP) marker development [14,19–22] and for other applications [23], including phylogenetic analysis [24] and adaptation studies [25].

To identify genes in new species, finding sequence similarity with published cDNA, EST or protein sequences in public database is now a widely accepted approach. Reliability of identification of novel sequences as putative genes however, can vary depending on the database screened. Depending on the database screened and the sequence similarity, identification of novel sequence as putative genes is effective not only for cDNAs, ESTs and proteins but also for gDNA sequences (GSSs) [26]. In addition, the GSS approach to predict putative genes can be a powerful compliment to EST profiling because there is the possibility that expression of a gene maybe associated with a particular developmental stage and this may not be recognized in an EST library depending on the samples screened [22]. According to Strong and Nelson [22], the GSS approach can be more productive and efficient than an EST approach for gene identification because redundant mRNA sequences from highly expressed genes can be avoided. GSSs which have low similarity and short sequence lengths can be problematical however, for obtaining the exact sequence from expressed genes using BLASTx searches because predicting exact exon-intron boundaries is often difficult. GSSs however, that possess high similarity and long sequence lengths can provide a feasible approach for predicting putative gene functions and for identifying potential exon–intron boundaries. While the majority of SSR markers could be neutral markers, mainly coming from non-coding regions, some SSR markers developed in functional genes can still be useful for evaluating functional diversity [13,15,27]. Here we screened putative SSR motifs from a partial GSS dataset and developed microsatellite markers for *H. siamensis* using a 454 pyrosequencing approach. Since there is little information about GSS in *H. siamensis*, this study will provide a foundation for studying the biology of this species from a fisheries management perspective.

## 2. Results and Discussion

**Figure 1:**
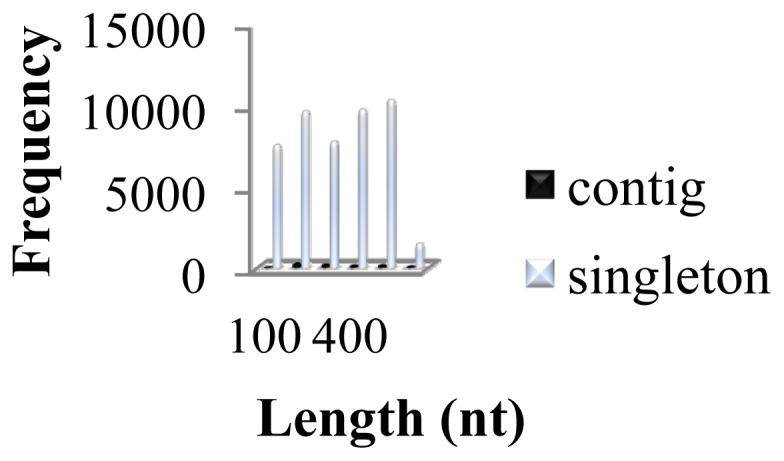
Summary of *H. siamensis* 454 pyrosequencing sequences.

Roche 454 GS-FLX sequencing and contig assembly of GSSs prepared from *H. siamensis* gDNA that was purified from fin tissue were undertaken. Sequences that passed basic quality standards were clustered and assembled *de novo*. The 454 sequencing run produced a total of 65,954 GSS sequences (total = 17.44 Mb) from gDNA isolated from pooled fin tissue samples. Average GSS length was 264 nucleotides (nt). Assembly of high quality GSSs generated 857 contigs averaging 352 nt in length from 5297 GSSs (Table 1). Most GSSs were unassembled as singletons comprising a total of 46,393 ESTs (total = 12.07 Mb) with an average GSS length of 274 nt. Total average GSS read length (264 nt) were longer than previous studies have reported in similar 454 sequencing runs for EST in non-model species including for Glanville fritillary (197 nt [19]), cichlid fishes (202 and 206 nt [25]) and European hake (206 nt [28]), but lower than for channel catfish (292 nt [29]), giant freshwater prawn (311 nt [21]) and bream (367 nt [14]). Different average read lengths may result from the total number of raw reads and different target material (mRNA *vs*. gDNA) subjected to sequencing. As shown in Figure 1, assembly of high quality *H. siamensis* GSS sequences generated 857 contigs varying in length from 101 nt to 2373 nt (average 352 nt; total 301,534 nt), with 143 (17%) being >500 nt in length. Singletons ranged from 50 nt to 742 nt in length (average 274 nt, total 12,706,536 nt) (Figure 1). To our knowledge, this is the first large scale study of genomic data from *H. siamensis*.

### 2.1. Comparative Analysis of GSSs

**Figure 2:**
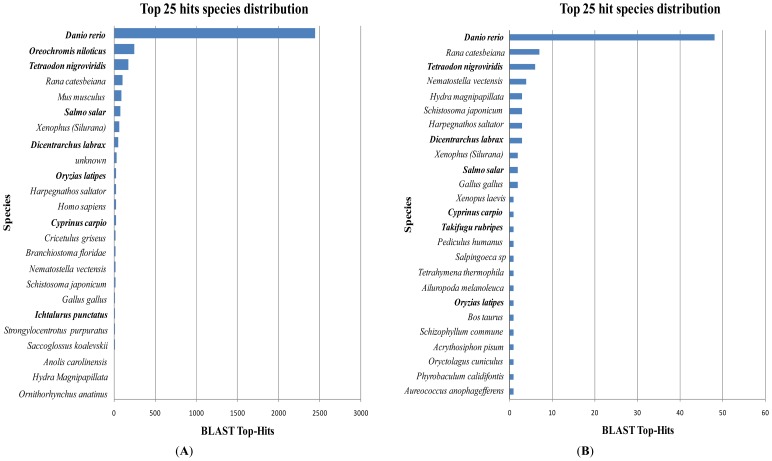
Top 25 hit species distribution based on BLASTx. *E* value cut-off is 10^−5^. Singleton (**A**) and contig (**B**). Bold text indicates teleosts.

BLASTx searches of *H. siamensis* GSS sequences showed that 90 of the 857 (10.5%) contigs and 3751 of the 46,393 (8.1%) singletons possessed significant similarity (*E* value < 10^−5^) with proteins in the GenBank non-redundant (nr) database (Table S1). The majority of contigs (62%) and singletons (87%) matched well to reported fish sequences (Figures 2a,b), an outcome that agrees with previous fish studies [20,25,30,31]. Species most represented in the BLASTx searches included zebra fish (*Danio rerio*), spotted green puffer (*Tetraodon nigroviridis*), European seabass (*Dicentrarchus labrax*), carp (*Cyprinus carpio*), Torafugu (*Takifugu rubripes*), medaka (*Oryzias latipes*), Nile tilapia (*Oreochromis niloticus*), Atlantic salmon (*Salmo salar*), Florida lancelet (*Branchiostoma floridae*), and channel catfish (*Ictalurus punctatus*), largely due to their availability of their sequences in public databases. Sequence homologies are indicative of close evolutionary relationships of *H. siamensis* with other fish. While zebra fish (*Danio rerio*) produced the top hit number with *H. siamensis*, this does not necessarily imply that zebra fish is more closely related phylogenetically to *H. siamensis* than common carp (*cyprinus carpio*). The explanation may simply be that the sequence dataset available for zebra fish is greater than for common carp and that *H. siamensis* is close to other teleosts, in general. No GSSs identified here matched sequences published for *H. siamensis* in the database and this is most likely due to the low number of *H. siamensis* sequences currently available in the NCBI database (only a few mitochondrial DNA sequences were available [6,7]). The *H. siamensis* GSS sequences generated here will vastly expand the number of genes identified in this species. A significant number of *H. siamensis* GSSs did not possess coding sequences matching to any sequence in the GenBank database which is not surprising in uncharacterized GSS studies [21,25,29,31]. While most of the anonymous EST sequences probably span non-coding regions of gDNA, or are the result of assembly errors from homopolymers as reported in other EST and 454 pyrosequencing analyses [21,32,33], some may also constitute novel genes unique to this species or may not have been identified previously in teleosts.

### 2.2. Gene Ontology Assignment

**Figure 3:**
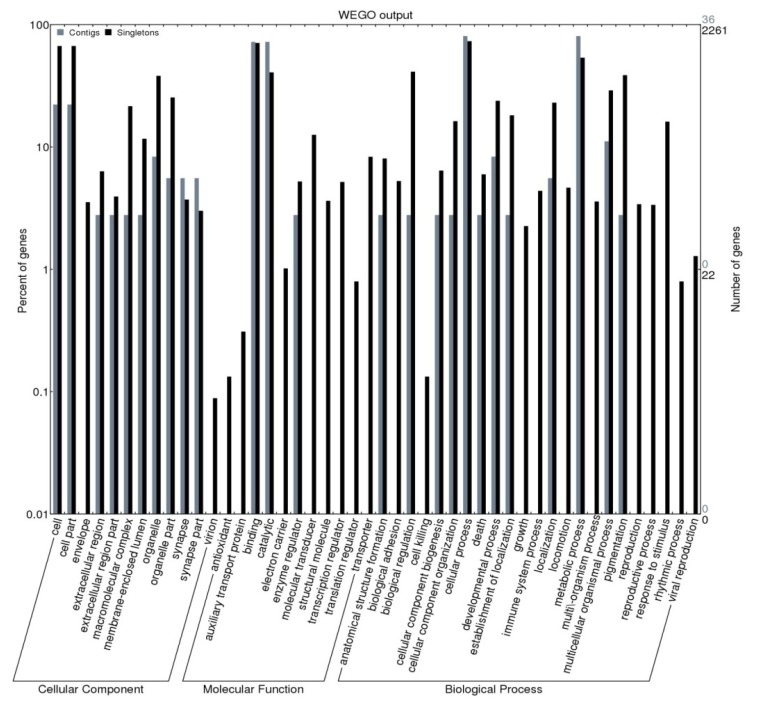
Gene Ontology (GO) terms for contig and singleton sequences in *H. siamensis*.

Gene Ontology (GO) terms could be assigned to 47,250 *H. siamensis* contigs (857) and singletons (46,393) based on BLAST matches to proteins with known functions (Figure 3, Table S1). GSS coding sequences were assigned to cellular components (4004 sequences), molecular function (4790 sequences) and biological processes (8313 sequences) (Figure 3). Among the *H. siamensis* GSSs assigned molecular functions, many were assigned to binding or catalytic functions, predominantly either enzyme regulators or molecular transducers (Figure 3). Cellular component assignments showed many GSS coding sequences were likely to possess cell and cell part functions, while those assigned biological process were mostly predicted to be involved in cellular or metabolic processes. Analyses of sequences in other fish species have identified transcripts possessing a similar range of potential metabolic functions [20,30,31].

### 2.3. KEGG Analysis

Many of the sequences present in the *H. siamensis* GSS contig and singleton dataset were identified to occur in KEGG pathways; purine metabolism (*n* = 49), phosphatidylinositol signalling system (*n* = 23), inositol phosphate metabolism (*n* = 17), oxidative phosphorylation (*n* = 15), and glycerophospholid metabolism (*n* = 14) (Table S1). Purine metabolism, that is important in numerous biochemical and cellular processes during vertebrate embryonic development [34], showed the highest number of GSSs here. During times of cellular stress (e.g., oxygen depletion or oxidative stress), purine metabolic pathways are involved in interactions with ATP and GTP levels [35,36] and mutations in these pathways can result in developmental defects including; eye-pigment disorders, eye-growth, bristle, and pupal lethality [34,37]. A total of 23 and 17 sequences were identified as related to phosphatidylinositol signalling systems and inositol phosphate metabolism, respectively. As one of the signalling molecules activated during oocyte and/or gonad maturation and activation of gonadotropin hormone release, phosphatidylinositol 3-kinase (Pik3) and inositol phosphate signalling systems have been observed in many species [38–42].

Of interest however, was that we recovered a high number of sequences that mapped to oxidative phosphorylation that are involved in ATP production in mitochondria [43,44]. Glycerophospholid metabolism, that plays a central role in the structure of cell membrane bilayers [45], also showed a high number of sequences recovered in *H. siamensis*. A total of 12 *H. siamensis* GSSs were predicted to be involved in the fatty acid metabolism pathway. Fatty acids are regarded to be key sources of metabolic energy in growth, reproduction and movement [45,46] and are important factors in maintaining homeostasis [46,47]. Although not all of the major genes reported in putative KEGG pathways were identified in *H. siamensis* GSSs, those that were detected provide insights into the specific responses and functions involved in molecular processes during *H. siamensis* metabolism.

### 2.4. Protein Domains

InterProScan searches identified 4015 protein domains among the 47,250 *H. siamensis* contigs (857) and singletons (46,393) (Table S1). Consistent with similar analyses in other fish species [20,30], domains that dominated include reverse transcriptase, immunoglobulin, integrase catalytic core, ribonuclease, and zinc finger domains (Table 2).

Immunoglobulin (Ig) motifs that play a critical role in the immune system where they recognize and respond to a wide range of antigens [48–50] were identified among the *H. siamensis* sequences, in particular, Ig-like fold (84), Ig-like (26), Ig V-set (24), and Ig I-set (14). Ig-like fold domains are also reported to be involved in a variety of functions including cell-cell recognition, cell-surface receptors, muscle structure and the immune system [51], and are often involved with protein-protein interactions mediated by their β-sheets as with other Ig-like domains [51,52]. In addition, the most common DNA binding motifs that present as transcription factors in a wide variety of organisms [53] were prevalent among the *H. siamensis* sequences, with 52 C2H2 and 31 C2H2-type zinc finger (Znf) domains identified. Transcription factors usually contain several Znf domains capable of making multiple contacts with DNA [54], and can also bind to RNA and protein targets [55].

23 domains containing integrase (IN) catalytic core were also predicted in the *H. siamensis* GSS sequences. IN is the virus-encoded enzyme responsible for key catalytic events associated with integration [56,57]. A total of 15 cadherin families that are involved in mediating calcium dependent cell-cell adhesion as transmembrane glycoproteins and that are crucial to various steps during embryonic development, were also predicted [58,59]. Other common domains identified in the dataset include; GPCR rhodopsin-like 7TM, Protein kinase catalytic domain and Protein kinase-like.

### 2.5. Analysis of Genes

Among GSSs derived from contig sequences with homology to ORF2-encoded protein, novel protein, reverse transcriptase-like protein, transposase, and enzymatic poly were most abundant (Table 3). In addition, among GSSs derived from singleton sequences with homology to retrotransposable element tf2, novel protein, ORF-encoded protein, reverse transcriptase-like protein, and transposase were most abundant. GSSs detected commonly in both contig and singleton sequences included ORF2-encoded protein, novel protein, reverse transcriptase-like protein, transposase, enzymatic poly, and protein nlrc3-like (Table 3). Although the focus of the current study was mainly to identify putative SSR motifs in the *H. siamensis* genome, several putative functional sequences identified provide a foundation for future genetic studies. GSS sequences with identified putative functions provide a starting point for deciphering the potential role of novel genes in each tissue, but further studies will need to be conducted to understand the molecular basis of specific genes.

Transposable element tc1 transposase, identified in the *H. siamensis* GSS dataset belongs to a superfamily of class-II transposable elements (TEs) widely present from protozoa to vertebrates [60,61] and it has also been identified in several teleost species [62–64]. As the name suggests, it has been hypothesized that the transfer of TEs from one genome to another potentially occur by vertical (sexual) or horizontal transmission [63]. Active TEs found in several fish have been reactivated successfully after molecular genetic manipulation from inactive genomic copies [65]. A larger number of TEs or mobile sequences could be useful for identifying genes important in fish aquaculture using inverted terminal repeats [65–67]. Furthermore, understanding the dynamics, control and evolution of fish TEs could allow insertion of selected sequences into fish germ cells to develop transgenics or for identifying genes important for growth and/or in somatic cells to improve DNA vaccination [65].

In the current study, we found a high occurrence of a gtpase IMAP family member 8-like sequence that was the first reported member of a family of putative GTPases but was renamed as GTPase in the immunity-associated protein family (GIMAP) [68]. GIMAP proteins are thought to be involved mainly in regulation of cell death in vertebrates [69] but a possible immune role has been suggested in Atlantic salmon and three-spine stickleback [70,71]. It is likely that GIMAP could provide a candidate molecular marker for immune studies in fish, where disease is a significant problem in many cultured species.

### 2.6. Putative Microsatellite Markers

**Figure 4:**
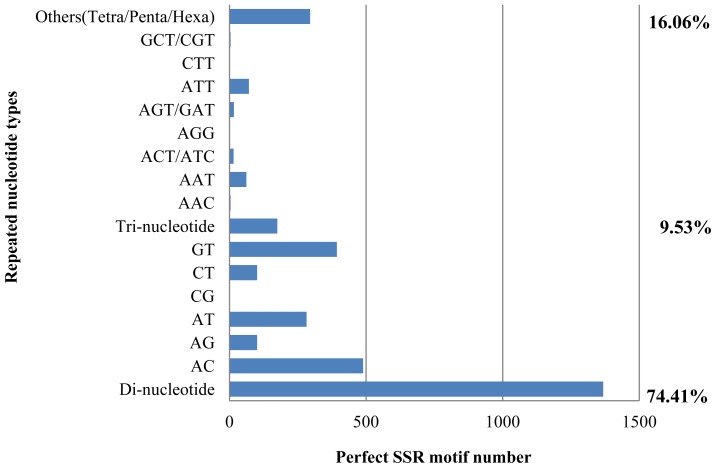
Distribution of simple sequence repeat (SSR) nucleotide classes among different nucleotide types in *H. siamensis*.

A total 1837 simple sequence repeats (SSRs) or microsatellites comprising 74.41% dinucleotide repeats, 9.53% trinucleotide repeats and 16.06% tetra/penta/hextanucleotide repeats were detected (Figure 4) among the *H. siamensis* GSS sequences. Recognition of a high number of dinucleotide repeats in *H. siamensis* is consistent with previous studies in fish and other aquatic organisms [21,14]. From 1837 detected SSRs, a total of 422 SSR primer sets comprising 78% dinucleotide repeat primers, 8.3% trinucleotide repeat primers, and 13.7% tetra/penta/hextanucleotide repeat primers were designed successfully (Table S2). The majority of SSRs detected and primers designed for their amplification in *H. siamensis* were singletons. A large number of SSRs were detected in *H. siamensis* contig sequences but primers were not designed successfully in these sequences. This result suggests that the GSSs in *H. siamensis* mostly consisted of non-coding regions which have high polymorphic sites, or alternatively this could be a common homopolymer problem with 454 sequencing.

A large number of PCR primers were designed from predicted polymorphic SSRs (Table S2) and await validation as genetic markers for examining adaptive and ecological processes in *H. siamensis* as has been done with other non-model species [e.g., 31,14]. In addition, SSRs detected here are potentially transferable to other closely related teleost species [14,72–74]. The potential markers identified here in *H. siamensis* GSSs will provide a valuable resource for studying the evolution and ecology of this species and can be applied to genome mapping and quantitative trait loci (QTL) analyses.

### 2.7. GSS-SSR Markers Test

One thousand eight hundred and thirty seven sequences containing SSR motif repeats were identified, of which, 422 qualified for primer design. At present, CAG and GATA repeat types predominate in vertebrate SSRs while common SSR markers developed for fish genetic studies are dinucleotide (CA) repeats [73]. The most common repeat motifs in *H. siamensis* included AC, GT (dinucleotide repeat), AAT, ATT (trinucleotide repeat), AGAT and ATCT (tetranucleotide repeat). Saarinen and Austin [75] found the same common repeat motif set (AC, GT, AAT, ATT, AGT and ATCT) in a north American fish (*Esheostoma okaloosae*), while GATA and CAG repeat were the most common repeat motifs reported in Japanese flounder (*Paralichthtys olivaceus*) and silver crucian carp (*Carassius auratus gibelio*) [73,76].

We chose 25 tetranucleotide repeats for screening in *H. siamensis* based on their relative mutability compared with other repeat types [77] and their ease of scoring [78]. Eight loci were selected for subsequent polymorphism tests in two wild populations of *H. siamensis* samples (Battambang (BB), Cambodia and Ubon Rathachani (UB), Thailand). From the statistical results (Table 4), observed (*H*_o_) heterozygosity (mean ± standard deviation) ranged from 0.341 to 0.976 (mean 0.75 ± 0.04) while expected (*H*_e_) heterozygosity ranged from 0.504 to 0.962 (mean 0.83 ± 0.04). Number of alleles (*N*_a_) ranged from 6 to 37 (mean 17.44 ± 2.37) with a higher number of alleles detected per locus (6 out of 8 loci) in the UB population compared with the BB population.

BLASTx searches using the non-redundant (nr) database is a common approach for identifying functional roles of genes. After checking with BLASTx, only six out of eight SSR sequences generated from contigs in *H. siamensis* were identified as putative genes but most SSR sequences showed very low similarity (>9.00 × 10^−8^) except for H21 that was identified as trafficking protein particle complex 10 (4.00 × 10^−23^). It has been suggested that the Roche 454 platform can be useful for generating large fragment gDNA sequences to provide a wealth of anonymous nuclear loci markers in non-model organisms [79]. Therefore, it is likely unidentified contig sequences (for primers HS2 and HS12) could be new genes in this species or result from non-coding regions for which we were not able to find a putative function due to the limited genetic information currently available for the target species. A cautious interpretation must be made however, because of the complexity of gene structure and potential for detection of pseudogenes.

## 3. Experimental Section

### 3.1. Samples and DNA Extraction

*H. siamensis* samples were collected from fin tissues. Initial species identification was verified after examination of external morphological traits in the field and this confirmed via mtDNA sequencing. Fresh fin tissues stored in 95% ethanol were used for gDNA extraction using a modified salt extraction method [80].

### 3.2. Library Construction and 454 Pyrosequencing

Pooled gDNA sample from two individuals were sent to the Australian Genome Research Facility (AGRF), Brisbane, Australia, and subjected to 454 GS-FLX sequence analysis [81]. Randomly sheared gDNA yields were quantified using a Quant-iT RiboGreen fluorometer (Invitrogen, Mulgrave, Australia) and average lengths were determined by analysis of aliquots (1 μL) on a Bioanalyzer (Agilent, Mulgrave, Australia). Pooled gDNA samples were subjected to sequencing on a eighth of a pico-titer plate via 454 GS-FLX using pyrosequencing chemistry (Roche, Branford, CT, USA) according to the manufacturer’s protocol.

### 3.3. Sequence Cleaning and Assembly

All sequence reads taken directly from the 454 GS-FLX sequencer were run through the sff file program (Roche) to remove sequencing adapters A and B, poor sequence data and barcodes. Sequences containing homopolymers of a single nucleotide comprising >60% of the read and that were >100 nucleotides in length were discarded. Trimmed sequences were assembled *de novo* using the default parameters in Newbler 2.5.3 (Roche). After initial quality filtering, AGRF provided assembled contig and singleton datasets for analysis. Due to the small sample numbers subjected to 454 GS-FLX, SNP discovery was not conducted. All *H. siamensis* partial GSS sequences were submitted to NCBI Sequence Read Archive under Accession No. SRA 053105.

### 3.4. Annotation

BLASTx searches [82] of the GenBank non-redundant (nr) database hosted by the National Center for Biotechnology Information (NCBI) [83] were performed on all contigs and singletons to identify putative gene functions (*E* value threshold < 1 × 10^−5^). The Blast2GO software suite [84] was used to predict functions of individual GSSs, to assign Gene Ontology terms (The Gene Ontology Consortium 2008), and to predict metabolic pathways using Kyoto Encyclopaedia of Genes and Genome (KEGG) [85]. To identify protein domains, all GSS sequences were interrogated against the InterPro databases [86] using the InterProScan tool [87]. The numbers of contigs annotated with each GO term were quantified using WEGO [88].

### 3.5. Identification of GSS-SSR Motifs

All GSS sequences were searched for SSR motifs using Msatcommander [89]. Default settings were employed to detect perfect di, tri, tetra, penta, and hexanucleotide motifs (including compound motifs). To be assigned, dinucleotide SSRs required a minimum of eight repeats, and all other SSR types a minimum of six repeats. Maximum interruption between two neighboring SSRs to be considered a compound SSR was set at 100 nucleotides. Perl script modules linked to the primer modeling software Primer3 [90] were used to design PCR primers flanking each unique SSR region identified.

### 3.6. Microsatellite Screening, Amplification and Testing

SSR marker amplification can produce nonspecific bands if primers are located in flanking regions that grouped into similar sequence. To check the possibility of grouping, all sequences containing SSR motifs were detected using microfamily software [91]. Twenty five selected primers to be tested here were derived only from the unique flanking sequence. Initial primer tests for PCR amplification were performed on 10 individuals derived from five discrete populations from the Mekong River, and from this, eight primers were selected for further analysis. PCR reactions were conducted in 12.5 μL total volume containing 1.5 μL 5× My*Taq* Red Buffer (Bioline (Aust) Pty. Ltd Australia), 0.1 μL My*Taq* DNA Polymerase (Bioline (Aust) Pty. Ltd Australia), 0.4 μL (10 pmol) of forward and reverse primer and 1 μL of DNA template and ddH_2_O up to 12.5 μL. PCR conditions were 5 min at 94 °C, followed by 30 cycles of 30 s at 94 °C, 15 s at 57 °C and 15 s at 72 °C, then 5 min of 72 °C and 15 min of 15 °C. Multiplexed PCR products (1 μL of each locus) were then analyzed in an ABI-3500 sequencer for genotyping and 1 μL of GeneScan™ 600 LIZ^®^ Size Standard v2.0. GeneMapper software (Version 4.1; Applied Biosystems: Mulgrave, Australia, 2011) was utilized for allele scoring. Polymorphic loci were tested on 89 individuals from 2 populations, BB and UB, because these are the largest population samples available to us from the MRB and more variation is expected. The BB site is located in Cambodia (13°04′N; 103°08′E) while the UB site is located in Thailand (15°15′N; 104°52′E). GenAlEex 6 software [92] and Excel-microsatellite-toolkit version 3.1 [93] were used for statistical analyses. Presence of genotyping errors were checked using MICROCHECKER software [94] employing a 95% confidence level.

## 4. Conclusions

The pyrosequencing method using partial GSS sequences was applied to development of SSR markers for *H. siamensis*, an economically important species in the Mekong River Basin. To date, eight polymorphic SSR loci have been selected and tested for genetic studies in *H. siamensis* populations and a large number of potentially useful markers await validation in this species, of which many could have potential applications in closely related species. A large number of putative SSR markers provide new possibilities for studying genetic variation, phylogeography and population structure in the target species. In addition, several putative genes that could have immune and growth-related functions in *H. siamensis* were identified but require confirmation due to the complexity of nuclear gene structure. The methods described here allowed us to obtain a large number of SSRs rapidly and the markers will be useful for future management of wild *H. siamensis* populations and permit a better understanding of discrete wild population structure to be developed at different geographical scales across the MRB.

## Figures and Tables

**Table 1:** Summary of 454 pyrosequencing.

Description	Dataset
Total number of bases (Mb)	17.44 Mb
Average read length (nt)	264 nt
Number of reads	
Total reads	65,954
Assembled	5,297
Singleton	46,393
Repeat	280
Number of contig	
Total contigs	857
Average contig read length (nt)	352 nt
Largest contig (nt)	2,373 nt
Number of large contigs > 500 nt	143

**Table 2:** Summary of the top 20 domains combining contigs (Con) and singletons (Sing) in *H. siamensis*.

IPR accession	Domain name	Domain description	Total of occurrence (Con/Sing)
IPR006130	Asp/Orn_carbamoylTrfase	Aspartate/ornithine carbamoyltransferase	1 (1/0)
IPR006132	Asp/Orn_carbamoyltranf_P-bd	Aspartate/ornithine carbamoyltransferase carbamoyl-P binding,	1 (1/0)
IPR002126	Cadherin	Cadherin	15 (0/15)
IPR005135	Exo_endo_phos	Endonuclease/exonuclease/phosphatase	1 (1/0)
IPR001845	HTH_ArsR_DNA-bd_dom	HTH arsR-type DNA-binding domain	1 (1/0)
IPR013098	Ig_I-set	Immunoglobulin I-set	14 (1/13)
IPR007110	Ig_like	Immunoglobulin-like	26 (0/26)
IPR013783	Ig-like_fold	Immunoglobulin-like fold	84 (2/82)
IPR013106	Ig_V-set	Immunoglobulin V-set	24 (0/24)
IPR001584	Integrase_cat-core	Integrase, catalytic core	23 (1/22)
IPR011009	Kinase-like_dom	Protein kinase-like domain	14 (0/14)
IPR000719	Prot_kinase_cat_dom	Protein kinase, catalytic domain	15 (0/15)
IPR012337	RNaseH-like	Ribonuclease H-like	29 (1/28)
IPR000477	RVT	Reverse transcriptase	37 (2/35)
IPR000276	7TM_GPCR_Rhodpsn	GPCR, rhodopsin-like, 7TM	18 (0/18)
IPR002492	Transposase_Tc1-like	Transposase, Tc1-like	2 (2/0)
IPR002035	VWF_A	Von Willebrand factor, type A	2 (2/0)
IPR006612	Znf_C2CH	Zinc finger, C2CH-type	1 (1/0)
IPR013087	Znf_C2H2/integrase_DNA-bd 5	Zinc finger, C2H2-type/ integrase, DNA-binding	28 (0/28)
IPR007087	zf-C2H2	Zinc finger, C2H2	52 (0/52)

**Table 3:** Frequency of genes identified in contigs (Con) and singletons (Sing) in *H. siamensis*.

Candidate genes	*E* value range	Matched species	Length range (nt)	Total of occurrence (Con/Sing)
Enzymatic poly	3.12 × 10^−91^–4.80 × 10^−13^	*Tetraodon nigroviridis*	269–1037	15 (3/12)
Lrr and pyd domainscontaining protein 12	3.42 × 10^−35^–1.72 × 10^−8^	*Danio rerio*	123–439	19 (0/19)
Novel protein	1.18 × 10^−65^–5.42 × 10^−6^	*Danio rerio*	120–595	43 (7/36)
Orf2-encoded protein	4.63 × 10^−97^–3.33 × 10^−8^	*Danio rerio*	143–1679	27 (7/20)
Protein nlrc3-like	7.76 × 10^−71^–1.32 × 10^−9^	*Danio rerio*	220–528	13 (2/11)
Retrotransposable element tf2	3.95 × 10^−97^–4.63 × 10^−6^	*Takifugu rubripes*	145–527	120 (2/118)
Reverse transcriptase-like protein	7.74 × 10^−52^–1.34 × 10^−11^	*Danio rerio*	151–544	22 (4/18)
Sjchgc01974 protein	5.82 × 10^−27^–5.28 × 10^−7^	*Mus musculus*	139–430	18 (0/18)
Transposable element tc1 transposase 155 kda protein type 1-like	6.41 × 10^−23^–1.80 × 10^−8^	*Danio rerio*	128–233	5 (5/0)
Transposase	1.65 × 10^−47^–5.92 × 10^−12^	*Rana catesbeiana*	196–507	21 (4/17)

**Table 4:** Details of 8 tetranucleotide SSR repeats designed for *H. siamensis*.

Locus	Primer sequence	Repeat Motif	Pop *	*N*_a_ *	*H*_o_ *	*H*_e_ *	PIC *	PHWE *	Percent missing
HS2	GTGGCGGAAATGGGCTTC	(ATCT)^14	BB	15	0.868	0.913	0.907	0.602	7%
	CCTGAGGCATTTCATAAACTCCG		UB	18	0.619	0.902	0.889	0.000	10%
HS4	CTCATCACCCGCTGTGTTTC	(ATCT)^11	BB	35	0.775	0.962	0.961	0.006	0%
	CACACACTGACAGGCAGAC		UB	37	0.894	0.940	0.938	0.125	0%
HS5	TGTCGTTCTCTGGCTGTCC	(ATCT)^13	BB	23	0.976	0.932	0.928	0.081	0%
	CCCAGATACAGGAGTGGGATG		UB	19	0.787	0.919	0.913	0.078	0%
HS12	TTGCCTGGAGGACAAGACC	(ATCT)^9	BB	22	0.725	0.936	0.932	0.003	0%
	TGCCACTGCACAGTAAACG		UB	27	0.711	0.954	0.952	0.001	0%
HS14	ACACGAGTGAGGAGTGCTG	(CTGT)^9	BB	14	0.806	0.846	0.832	0.608	12%
	AGGCCACAAACTTCTGCTTG		UB	15	0.810	0.822	0.812	0.550	10%
HS21	CAACAAGCAGAGCGACAGG	(ACTC)^8	BB	7	0.730	0.705	0.657	0.981	0%
	TGTTGATAACGCGCCACAG		UB	11	0.596	0.750	0.722	0.569	0%
HS23	TGAATGGAATGAGAGGTTCAGC	(GAGT)^8	BB	12	0.878	0.830	0.810	0.303	0%
	TGCTGCTTGTGTGTTCAAAG		UB	13	0.957	0.873	0.860	0.000	0%
HS24	AACACCATACACCTGCACC	(AAAC)^8	BB	6	0.341	0.504	0.477	0.007	9%
	ACTCCTGTGGTGGAAGAAAGG		UB	5	0.467	0.528	0.483	0.000	0%

* Pop, population; BB, Battambang (41 samples); UB, Ubon Rathanchani (48 samples); *N*_a_, number of alleles; *H*_o_, observed heterozygosity; *H*_e_, expected heterozygosity; PIC, Polymorphism information content; PHWE significant at *p* < 0.003 after Bonferroni correction.
